# Hybrid Adhesive Hydrogel Patch Containing Genipin-Crosslinked Gelatin–Hyaluronic Acid for Future Use in Atopic Dermatitis

**DOI:** 10.3390/jfb16060195

**Published:** 2025-05-26

**Authors:** Nurul Ain Zawawi, Manira Maarof, Nur Izzah Md Fadilah, Daniel Looi Qi Hao, Yasuhiko Tabata, Mh Busra Fauzi

**Affiliations:** 1 Department of Tissue Engineering & Regenerative Medicine, Faculty of Medicine, Universiti Kebangsaan Malaysia, Cheras, Kuala Lumpur 56000, Malaysia; p143394@siswa.ukm.edu.my (N.A.Z.); manira@ukm.edu.my (M.M.); izzahfadilah@ukm.edu.my (N.I.M.F.); 2 Advance Bioactive Materials-Cells UKM Research Group, Universiti Kebangsaan Malaysia, Bangi 43600, Selangor, Malaysia; 3 My Cytohealth Sdn Bhd, Hive 5, Taman Teknologi, MRANTI, Bukit Jalil, Kuala Lumpur 57000, Malaysia; dr.daniellooi@cytoholdings.com; 4 Cell Biotechnology Group, Department of Plastic and Reconstructive Surgery, Graduate School of Medicine, Kyoto University, South Research Bldg. No.1 (Institute for Life and Medical Sciences) 53 Kawara-cho Shogoin, Kyoto 606-8507, Sakyo-ku, Japan; tabata.yasuhiko.7n@kyoto-u.ac.jp

**Keywords:** hydrogel patch, natural biomaterial, gelatin, hyaluronic acid, genipin, atopic dermatitis, wound healing

## Abstract

Hydrogel patches have gained significant attention in wound healing applications as they are similar to hydrogel dressings due to their moisture-retentive properties, biocompatibility, and ability to promote tissue regeneration. In this study, gelatin-based hydrogels crosslinked with genipin and incorporated with hyaluronic acid (HA) were developed to enhance mechanical stability, swelling behavior, and structural integrity. Fourier transform infrared (FTIR), thermogravimetric (TGA), and energy-dispersive X-ray (EDX) analyses were conducted and confirmed successful crosslinking and good thermal stability, ensuring hydrogel durability under physiological conditions. The optimized hydrogel (GE_HA_GNP) exhibited a sufficient water vapor transmission rate (WVTR), swelling ratio, and contact angle, allowing for effective wound exudate absorption and hydration maintenance, which is essential for accelerated healing. The findings demonstrate that the crosslinked hydrogels were able to maintain a WVTR of 500 to 1500 gm^−2^ day^−1^, a contact angle of >40°, and a swelling ratio of 700–1000%. The combination of genipin as a crosslinker and the addition of HA significantly improved the mechanical properties and biocompatibility of the hydrogels, making them promising candidates for an alternative treatment for atopic dermatitis and a potential wound dress-ing. Furthermore, the hydrogel patches show potential for future drug delivery appli-cations, with further studies required to evaluate their antimicrobial properties and long-term clinical performance.

## 1. Introduction

The skin, being one of the most important and largest organs of the body, is perfectly designed and created to shield us from environmental hazards, such as physical injuries, severe wounds, burns, and other traumatic injuries. Damage to the skin impairs the skin barrier’s line of defence and exposes the individual to a series of health issues depending on the severity of the injury. The four phases of wound healing—hemostasis, inflammation, proliferation, and remodelling—must occur to restore and heal the skin’s integrity and function. Any disruption during these phases may lead to chronic wounds and pose significant challenges in a clinical setting. However, the slow wound healing process can be notably slower in skin conditions like atopic dermatitis, one of the most common chronic inflammatory skin conditions, wherein consistent inflammation and barrier failures are present [1]. It possesses several factors such as microbial colonization, prolonged inflammation, and an increase in transepidermal water loss, which all disrupt the wound healing process and increase the risk of chronic wounds. It also has a constant “itch-scratch” cycle that leaves the skin vulnerable to itching as well as increasing the inflammation and reactions towards any mechanical or chemical stimulation [2]. If left untreated, the barrier function of the skin weakens, making it more susceptible to dehydration and infection [3].

The complexity of the wound healing process and the risk of chronicity occuring have made wound healing a key focus in the field of tissue engineering and regenerative medicine. A moist wound environment is needed to accelerate the wound healing process by promoting keratinocytes’ migration over the wound area [4,5], especially for the treatment of atopic dermatitis as there is an imbalance in keratinocytes. Thus, wound dressings are commonly used to establish and regulate a moist environment for the wound. Traditional wound dressings such as gauze, foams, hydrocolloids, and films are commercially available and are widely used by clinicians; however, they often lack functional properties such as flexibility, adhesion, and moisture retention, which are important for exudate absorption, similar to treatments for atopic dermatitis, wherein the side effects often outweigh the advantages of current-based treatments such as steroidal creams.

Despite these advancements, achieving an effective, high-quality, and quick wound application continues to be a pressing challenge for both clinicians and researchers. The challenge further increases when the rapid market growth of global wound care products is estimated to reach USD 18.7 billion by the year 2027 from USD 12 billion in 2020 [6,7]. This may be due to the ageing population and other underlying conditions such as diabetes. To combat this rising problem, numerous innovative studies have been conducted to enhance the wound healing process using biomaterials and nanotechnology while also reducing the risk of complications.

In this study, we propose the use of a simple but effective hydrogel patch as an alternative to current wound healing dressings. Hydrogels are a three-dimensional polymeric network that are known for their ability to retain moisture and good biocompatibility and biodegradability while also ensuring the delivery and controlled release of active components [8]. The main polymers used for this hydrogel patch are all-natural based, consisting of gelatin, hyaluronic acid, and genipin.

First of all, gelatin, derived from denatured collagen, is commonly used in hydrogels due to its ability to promote cellular proliferation, migration, adhesion and biodegradation [9]. It is also non-toxic, easily accessible, and low in cost, making it a highly suitable material for biomedical applications [10]. Next, the addition of hyaluronic acid, which is widely used in skin care products and more, is known to enhance the activation of components such as macrophages, fibroblasts, and collagen [11]. Moreover, HA has favourable hyper elasticity, strength, and biocompatibility for biomaterial applications but weak mechanical properties and rapid degradation in hydrogels [11]. Therefore, the addition of a crosslinker is necessary to enhance its mechanical properties. In this study, genipin was chosen for the crosslinker of hydrogels; it is derived from the fruit of *Gardenia jasminoides*. It is known for its biocompatibility, low toxicity, and ability to enhance the mechanical stability of biomaterials by forming stable covalent bonds with amino groups in proteins [12]. Due to these properties, genipin is widely used in biomedical applications, including hydrogel fabrication, tissue engineering, and drug delivery systems.

The aim of this study is to develop and characterize a topical hydrogel patch using natural polymers, such as gelatin, hyaluronic acid, and genipin, to create an advanced wound dressing that focuses on dry and inflammatory skin conditions like atopic dermatitis that can improve skin barrier function and support wound healing.

## 2. Materials and Methods

The study design was approved by Universiti Kebangsaan Malaysia Research Ethics Committee (Grant Code: FF-2025-062 and Ethics Approval Code: JEP-2024-990).

### 2.1. Preparation of Gelatin and Hyaluronic Acid-Based Hydrogels

**Figure 1:**
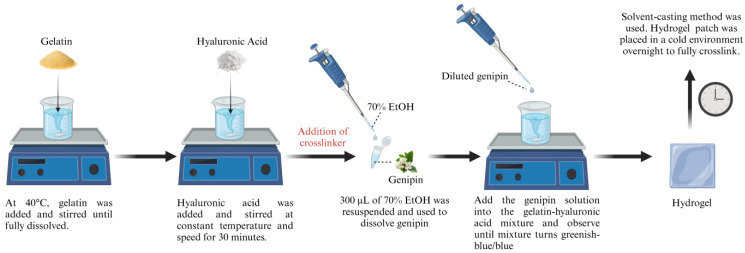
Schematic diagram of hydrogel patch preparation.

For the preparation of the hydrogel, gelatin, hyaluronic acid, and genipin as the crosslinker were used, of which the exact hydrogel compositions can be seen in Table 1 and Figure 1. A 9% (*w*/*v*) solution of gelatin (GE) powder (Nitta-Gelatin Ltd., Yao City, Osaka, Japan) was dissolved in distilled water (dH_2_O) at 40 °C using a hot plate stirrer. Once the gelatin was fully dissolved, 0.2% (*w*/*v*) of hyaluronic acid (HA, molecular weight: 200 kDa), which was provided in collaboration with Professor Yasuhiko Tabata from Kyoto University, was added to the gelatin mixture and stirred continuously at a constant temperature and speed. A solution of 0.1% (*w*/*v*) of Genipin (GNP) (FUJIFILM Wako Pure Chemical Corporation, Chuo-Ku, Osaka, Japan) was prepared through dissolution in 70% ethanol (EtOH; MERCK, Darmstadt, Germany) and added to the hydrogel mixture to obtain the final formulations of GE_GNP and GE_HA_GNP whereas the non-crosslinked hydrogels were represented as GE and GE_HA. The gelation time of the hydrogels was then recorded at ±27 °C while the hydrogel was in a gel state, using the inverted tube test method as it may provide valuable insights for the development of future bioinks and serve as a reference for potential mass production applications.

To create the hydrogel patch, the solvent casting method was used, wherein 3 mL of the hydrogel mixture was carefully poured into a silicone mold of 55 mm diameter, to obtain an estimated thickness of 1 mm [13], and stored in a chiller overnight to allow the hydrogels to fully crosslink and stabilize. The hydrogel was then removed from the mold, and patches were cut with a width of 25 mm. The prepared samples were then used for further analysis.

### 2.2. Gross Appearance of the Hydrogel Patches, Weight, Thickness, and Folding Endurance

The top and lateral views of the topical hydrogel patch, both crosslinked and non-crosslinked, was captured after 24 h of preparation using a digital camera (Nikon, Tokyo, Japan). For each group of hydrogel formulations, three random patches were individually weighed, and their thickness was measured using a vernier calliper to obtain the average measurements. The folding endurance was also observed manually based on a study [14] by folding the hydrogel repeatedly at the same location until visible cracks or tears were seen.

### 2.3. Adhesion Evaluation

The hydrogel patches were prepared and cut into uniform dimensions of 2 cm × 4 cm. The patches were manually assessed through a series of test designed to simulate practical applications. The adhesion and flexibility of the hydrogel patches were evaluated using simple manual tests, including bending, compressing, twisting, and curving, to mimic various real-world applications. For the bending test, the hydrogel patch was adhered to a flat substrate, and one corner was lifted and bent to a 90° angle to assess detachment or peeling. In the compression test, moderate pressure was applied to the patch using the palm to evaluate its ability to remain adhered under compression. The twist test involved twisting the patch on the substrate to examine its resistance to shear stress, while the curve test assessed adhesion on a curved surface, such as the back of the hand or a joint area, by observing lifting or wrinkling. These qualitative assessments provided insights into the hydrogel’s adhesion and mechanical properties under different deformation scenarios, enabling comparisons between crosslinked and non-crosslinked formulations.

### 2.4. Chemical Characterization of the Hydrogel Patches

#### 2.4.1. Fourier Transform Infrared (FTIR)

FTIR was employed to detect the different characteristic peaks of functional groups and analyse the structural relationships between components. The FTIR spectra of the natural polymers, crosslinker, and crosslinked and non-crosslinked hydrogel patches were executed at a resolution of 4 cm^−1^, using a Perkin Elmer spectrometer (Waltham, MA, USA). Before obtaining the spectra, freeze-dried hydrogels were finely crushed into powder using a pestle and mortar. The hydrogels’ functional groups and absorbance peaks were evaluated with the wavelength range of 4000 cm^−1^ to 500 cm^−1^.

#### 2.4.2. Simultaneous Thermal Analysis

To measure the thermal stability and physicochemical changes of the hydrogel patch and its pure components, thermogravimetric analysis (TGA) (model TGA-50, Shimadzu, Kyoto, Japan) was conducted. The dynamic tests were performed in a nitrogen-purged environment, with the temperature increasing from 25 to 800 °C at a constant heating rate of 10 °C/min. The weight loss of the freeze-dried hydrogel as a function of temperature was continuously recorded. The obtained results were then analysed using ta60w (v7.0) software.

#### 2.4.3. Energy-Dispersive X-Ray (EDX)

The elemental composition was also assessed via EDX to assess the elemental composition in the hydrogels using a Phenom Pro X SEM EDX microscope (Phenom, Eindhoven, The Netherlands). The carbon, oxygen, and nitrogen content of the hydrogel patches were analysed, and GE was used as the control.

### 2.5. Porosity Measurement

According to previous studies, two approaches can be used to assess the porosity percentage of hydrogels. Both methods use freeze-dried hydrogels and are complementary to each other for the analysis of a scaffold’s porous nature.

#### 2.5.1. Liquid Displacement Method

Initially, freeze-dried hydrogels were weighed before being immersed in 99.5% ethanol for 6 h. After 6 h, the hydrogels were reweighed, and the porosity was calculated using the following formula:

$$Porosity\left(\%\right)=\frac{\left(Wf-Wi\right)}{\rho V}\times 100$$

where Wf indicates the final weight of the hydrogel, Wi indicates the initial weight of the hydrogel, and ρ indicates the alcohol density (0.789 g/m^3^), which is then multiplied by the volume of the hydrogel.

#### 2.5.2. Scanning Electron Microscopy (SEM)

The SEM was operated at 15 kV to observe the morphological features of the hydrogel patches. Micrographs of the hydrogels were captured at magnifications of 500 and 1000×, and at high magnifications, a rough and porous surface was observed. To prepare for SEM analysis, the hydrogels were freeze-dried to create a lyophilized hydrogel, which was later coated with a nanogold layer using ion sputtering. The microstructure of the hydrogel network was then analyzed using field-emission scanning electron microscopy (FESEM) (Supra 55VP model, Jena, Germany), as described in a previous study [15,16]. This analysis provided a detailed high-resolution image of the hydrogels, specifically the pore size, distribution, and connectivity.

### 2.6. Swelling Ratio

The *swelling ratio* of the hydrogel was evaluated to assess the hydrogel’s capacity to absorb wound exudates, by using a method optimised from a previous study [17,18]. The swelling ratio was measured by weighing the initial weight (*Wi*) of a freeze-dried scaffold, which was later immersed in phosphate-buffered saline (PBS, pH 7.4) for 6 h at room temperature. After 6 h, excess PBS was removed using filter paper, and the final weight (*Wf*) was recorded. The ratio was then calculated using the following formula:

$$Swelling\;ratio\;\left(\%\right)=\frac{Wf-Wi}{Wi}\times 100$$

where *Wf* represents the final weight of the hydrogel after immersion with PBS, and *Wi* represents the initial weight of the hydrogel before immersion.

### 2.7. Contact Angle

The contact angle was determined to assess the hydrophilicity of polymerised hydrogel’s surface characteristics. A 10 µL drop of dH_2_O was dropped onto the surface of the hydrogel, and after 10 s, a image was captured and analysed using the ImageJ software (National Institute of Health, V1.5, Bethesda, MD, USA).

### 2.8. Water Vapor Transmission Rate (WVTR)

Following the American Society for Testing and Materials (ASTM) standards, the *WVTR* was evaluated to test the hydrogels’ ability to facilitate water evaporation and gas exchange, which are both important for the promotion of wound healing. The hydrogel was placed on top of a cylindrical glass bottle that was filled with 10 mL of dH_2_O and securely wrapped with parafilm. The setup was then incubated in an incubator at 37 °C in a controlled atmosphere with 5% CO_2_. The *WVTR* was then calculated using the following formula:

$$WVTR\;\left(\%\right)=\frac{\left(Wi-Wf\right)}{A\times Time}$$

where *Wi* represents the initial weight of the hydrogel and *Wf* represents the final weight of the hydrogel, both weighed with the cylindrical bottle, *A* is the area of the opening of the cylindrical bottle’s surface area, and Time represents the number of days it was incubated.

### 2.9. Mechanical Property Analysis

#### 2.9.1. Compression and Resilience

The hydrogels’ mechanical properties were measured by using a manually modified compression technique to assess their strength and suitability for tissue engineering applications [15]. Polymerized and cylindrical-shaped hydrogel samples were compressed using a 300 g metal load for 2 min, at room temperature, with a diameter of 25 mm and a height of 5 mm. Images of the scaffold were taken at eye-level before, during, and after compression using a digital camera (Nikon, Tokyo, Japan), and the area of thickness was observed and analysed in ImageJ software (version 1.54k, NIH, Bethesda, MD, USA). *Compression* and *resilience* were then calculated using the following formula:

$$Compression\;\left(\%\right)=\frac{Ac}{Ai}\times 100$$

$$Resilience\;\left(\%\right)=\frac{Af}{Ai}\times 100$$

where *Ai* represents the area of thickness before compression, *Ac* represents the area of thickness during compression, and *Af* represents the area of the hydrogel after compression.

#### 2.9.2. Tensile Strength Analysis

Tensile testing was conducted by using a 20 N load cell (Model UUK 5, Chungcheongbuk-do, Republic of Korea) equipped with a micro-stepper motor system (Ezi Step, Fastec, Bucheon, Republic of Korea) and an OMRON RXRX25 data logger to record the load. The elongation was determined using a 1.0 mW Omron laser detector with a detection limit of 2.5 ms/600 nm. The tensile strength was taken as the maximum stress of the stress–strain curve. The hydrogel samples were cut into a rectangular shape of 15 mm width, 40 mm length, and 2 mm thickness. The gauge length was constant at 15 mm in length and the thickness was measured using a vernier calliper. Sandpaper was glued onto the clamps to prevent the hydrogel from slipping, and the hydrogels were held between the clamps and pulled by the top clamp at a crosshead velocity of 3 mm/min. The elongation at break (ε) and tensile strength (σ) were measured when the hydrogels were torn apart. They were then calculated using Equations (1) and (2) based on the method described in [19]:

(1) $$\varepsilon \;\left(\%\right)=\frac{\Delta L\;(\mathrm{mm})}{L0}\times 100$$

(2) $$\sigma \;\left(\mathrm{kpa}\right)=\frac{Pmax\;(\mathrm{kN})}{A0\;(m^{2})}\times 100$$

where Δ*L* represents the deformation of the sample, *L0* is the initial sample length, *Pmax* is the breaking force, and *A0* is the cross-sectional area of the sample.

### 2.10. Statistical Analysis

The data were analyzed using GraphPad Prism version 10.4 (GraphPad Software, Inc., San Diego, CA, USA), OriginPro version 2025 (OriginLab Corporation, Northampton, MA, USA), and SigmaPlot Version 10.0 (Systat Software, Inc., San Jose, CA, USA). Statistical comparisons using one-way and two-way analysis of variance (ANOVA) were also made across the groups. The quantitative results were expressed as the mean ± standard deviation (SD), and the *p*-value of less than 0.05 was considered statistically significant. All of the experiments were performed in triplicate (n = 3).

## 3. Results and Discussion

### 3.1. Characterization of the Adhesive Hydrogel Patches

**Figure 2:**
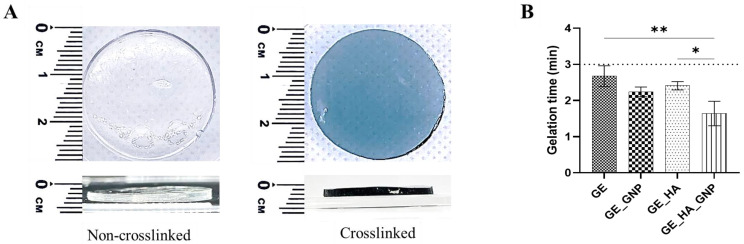
(**A**) Gross appearance (top and lateral views) of non-crosslinked (GE) and crosslinked (GE_GNP) hydrogels and (**B**) the gelation time for each group, where * indicates a significance of *p* < 0.05 and ** indicates a significance of *p* < 0.01.

Figure 2A shows the hydrogels’ gross appearance when chemically crosslinked using genipin (GNP); it includes the top and lateral views, with each being around roughly 1 mm in thickness to mimic the thickness of commercially available patches [13]. In terms of appearance, once the hydrogel has been polymerized and crosslinked with GNP, it will turn into a bluish-green-to-dark blue colour, signifying the success of forming a covalently crosslinked network, whereas non-crosslinked hydrogels are colourless and clear despite the addition of hyaluronic acid, indicating the absence of a crosslinker [18]. No visible differences in the appearance of the hydrogels (GE_HA and GE_HA_GNP) were observed when hyaluronic acid was incorporated. Instead, this observation aligns with a previous study that stated HA does not alter the macroscopic appearance but can enhance certain properties of the hydrogel [20]. The blue coloration is a reaction between the amine groups in gelatin and genipin, initiating a nucleophilic attack that opens genipin’s cyclic structure [9]. This reaction leads to the formation of intermediate aldehyde groups, which then react with additional amine groups to form a nitrogen-containing ring structure. Further interactions between the amine groups of gelatins and the ester groups of genipin result in the formation of stable amide bonds (-CONH-) [10]. These amide bonds both facilitates intra- and intermolecular crosslinking, strengthening the gelatin network, making it suitable for biomedical applications that focus on a more natural and biocompatible approach.

Moreover, Figure 2B shows the gelation time for different hydrogel formulations: gelatin only (GE), gelatin–genipin (GE_GNP), gelatin–hyaluronic acid (GE_HA), and gelatin–hyaluronic acid–genipin (GE_HA_GNP). The gelation time was observed within three minutes at 23–26 °C, initiated by a starting point of 27 °C. The results indicate that the addition of genipin in the GE_GNP formulation slightly reduced the polymerization time compared to GE. The addition of hyaluronic acid in GE_HA did not significantly affect the polymerization time, while the combination of materials in GE_HA_GNP resulted in the shortest duration of polymerization time. The fast polymerization time in GE_HA_GNP is likely due to genipin’s ability to form covalent bonds with amine groups in gelatin, enhancing crosslinking efficiency [21]. While gelation time is a critical factor for other applications such as 3D-bioprinting or in situ gelation, wound dressings and patches also prioritize structural integrity, biocompatibility, and the ease of large-scale production. The optimized hydrogel formulation (GE_HA_GNP) not only ensures a stable and uniform gel network but also facilitates mass production while maintaining consistency in mechanical properties and hydration levels.

### 3.2. Physical Characterization of the Adhesive Hydrogel Patches

To evaluate the physical properties of the hydrogel, the thickness, weight variation, and folding endurance of the hydrogel patch formulations were assessed. Based on Table 2, each formulation exhibited different but similar results in thickness and weight, wherein the weight varied from 0.46 to 0.53 g. Using a vernier calliper, the average thickness of all of the hydrogel patches ranged from 1.02 to 1.24 mm, which aligns with the typical thickness of commercially available wound dressings at 1.26 mm according to a previous study [13].

Folding endurance was also tested to a maximum of 350 folds each. This test helps to observe the flexibility of each hydrogel formulation. However, to avoid biases in strength and folding method, each group had a minimum of three individuals to minimise the bias in the assessment of folding endurance; a minimum of three different individuals conducted the folding tests simultaneously, each evaluating the patches independently and ensuring consistency in the measurements. It was observed that GE_HA_GNP (300 ± 10.31) exhibited the greatest folding endurance, followed by GE_GNP (233 ± 5.80), suggesting that the addition of hyaluronic acid and genipin as a crosslinker improves the flexibility of the hydrogel and resulted in it not being brittle. Nevertheless, although this method provides insight into the flexibility and durability of the hydrogel groups, especially when crosslinked, the manual procedure may influence the hydrogels’ behaviour, leading to variability in the results.

**Figure 3:**
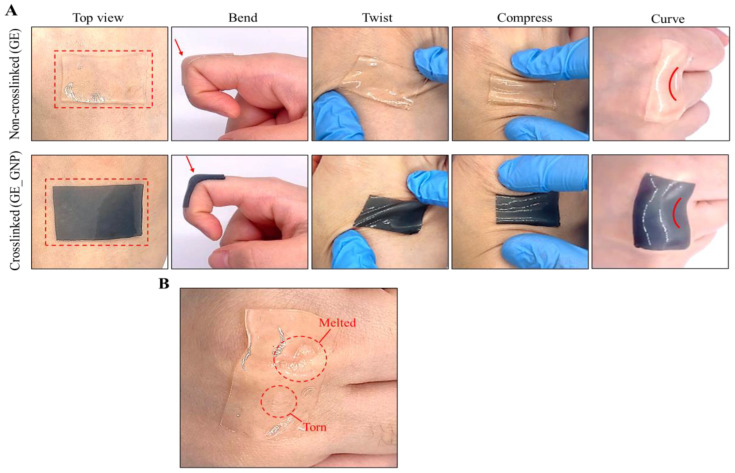
Characterization of topical adhesive hydrogel patches. (**A**) Representative image showing the adhesion of the GE and GE_GNP hydrogel patches attached to the back of the hand under various external stimuli, such as bending of the fingers and compressing, twisting, and curving of the hydrogel on knuckles. The red boxes indicated the area and appearance of application on the skin, the arrows indicate the direction of deformation or movement, and the wave line illustrates the curvature of the hydrogel on the skin. (**B**) Observation of the non-crosslinked (GE) hydrogel patch when placed onto the skin after 2 min, showing signs of melting and torn areas as marked.

Furthermore, the adhesive properties of the hydrogel patches were also evaluated to determine their ability to conform to any parts of the skin under various external stimuli, including the bending, compressing, twisting, and curvature of the hydrogel. Surprisingly, during the fabrication process of the hydrogel patch, it was discovered that hydrogels with a thickness of more than 2 mm were neither as adhesive nor as stretchable. This finding was then later supported by a study, stating that thinner hydrogels store less elastic energy, which allows the hydrogel to stretch more freely [22]. Additionally, elasto-capillary effects also play a crucial role in thin and soft hydrogels due to their ability to maintain stable surfaces and strong adhesion by increasing surface tension forces. This can be compared to thicker hydrogels that experience higher elasto-capillary lengths, often leading to a wrinklier and creasing appearance, as well as a reduction in adhesive performance [22,23]. As observed in Figure 3A, we provide a comparison of the GE (non-crosslinked) and GE_GNP (crosslinked) hydrogel patches applied to the skin, showing that they adhered well onto the skin while following the bending and curving of the skin. However, as shown in Figure 3B, the non-crosslinked (GE) hydrogel showed noticeable melting and tearing of the hydrogel after 2 min on the skin (body temperature: 37 °C). It also underwent poor degradation and durability and exhibited weak mechanical stability, as it deformed easily under stress. In contrast, crosslinked hydrogels were able to withstand the body temperature for a long period due to the formation of covalent bonds enhancing the three-dimensional network and mechanical properties [20].

### 3.3. Chemical Evaluation of the Hydrogel Patch

#### 3.3.1. Fourier Transform Infrared (FTIR) and Thermogravimetric Analysis

**Figure 4:**
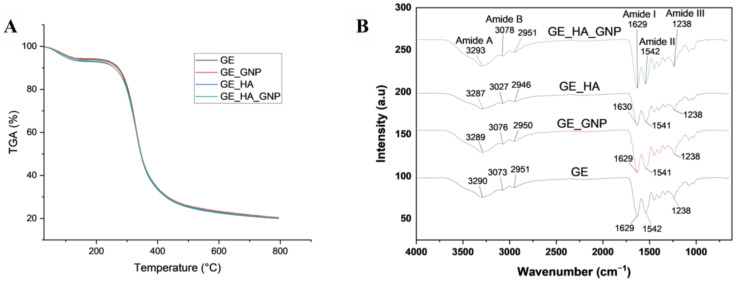
Chemical characterization of the hydrogel groups. (**A**) TGA analysis of the different hydrogel groups and (**B**) FTIR spectra labelled with the functional groups.

Focusing on the chemical characterization of the hydrogel patches, TGA and FTIR were analysed. TGA was conducted to provide insight into the thermal stability and degradation behaviour of the hydrogel patches, mainly focusing on mass loss and thermal degradation peaks. Based on Figure 4A, during the first phase of the initial moisture loss, all of the hydrogel patch groups exhibited similar initial mass loss, with that in GE and GE_HA occurring at 83 to 86 °C, with a mass loss of 5.8 and 7.1%. GE_GNP and GE_HA_GNP exhibited a slightly lower water loss between 83 to 90 °C, with a mass loss of 6.2 to 6.5%. During the second phase of polymer breakdown, GE_GNP and GE_HA_GNP exhibited higher degradation temperatures between 328 to 330 °C, with a mass loss of 73.4 and 73.2%. This may indicate that with the addition of genipin, the thermal stability slightly increased. However, GE and GE_HA exhibited lower degradation temperatures of 325 to 329 °C, with a mass loss of 74 and 73%. The mass loss during the third phase of the major decomposition of all hydrogels was similar.

#### 3.3.2. Dispersive X-Ray (EDX)

Furthermore, EDX was analysed to determine the elemental compositions inside the hydrogel patches, specifically carbon (C), oxygen (O), and nitrogen (N), as seen in Table 3. Slight differences can be seen in the results, providing insights into the effects of adding hyaluronic acid and genipin. The carbon element was dominant in both crosslinked and non-crosslinked groups, showing a slight increase from 45.3 ± 1.25 (GE) to 48.1 ± 1.36 (GE_GNP) % when genipin was incorporated into the hydrogel. However, with the addition of hyaluronic acid in the hydrogel, the carbon content remained within a similar range and did not significantly alter the carbon element in the hydrogel patch. Similarly, the oxygen element also displayed no significant values between each group. Other than that, for the nitrogen element, non-crosslinked hydrogels were observed to exhibit higher levels of nitrogen (22.4 ± 1.75% in GE and 21.0 ± 1.35% in GE_HA), whereas the incorporation of genipin as the crosslinker in the hydrogel patches reduced the levels of nitrogen (19.2 ± 1.93% in GE_GNP and 20.55 ± 1.45% in GE_HA_GNP). However, all hydrogel groups displayed no statistically significant changes in their elemental compositions, indicating the uniform incorporation of components throughout the network.

### 3.4. Micro-Morphological Evaluation of the Hydrogel Patches

**Figure 5:**
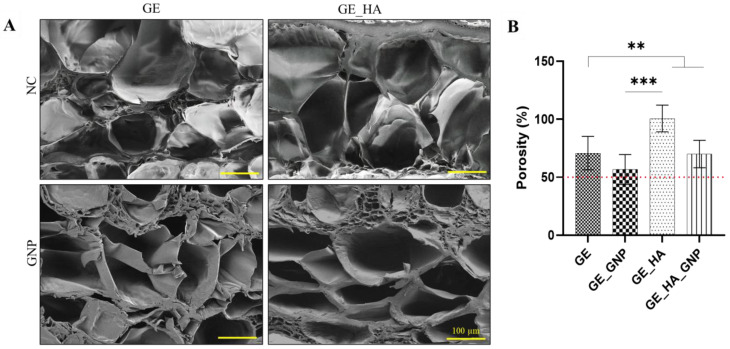
SEM analysis and cross-sectional view of the hydrogel patch. (**A**) SEM analysis of the different hydrogel groups under 100× magnification and (**B**) porosity at 24 h (%) using the liquid displacement method, where the dotted line indicates the targeted porosity value. ** indicates a significance of *p* < 0.01 an *** indicates a significance of *p* ≤ 0.001.

The microstructure of the patches was observed using SEM analysis, as shown in Figure 5A, showing the cross-sectional view of the different hydrogel patches at 100× magnification. It can be seen that the non-crosslinked patches (GE and GE_HA) appear to have larger, well-developed pore sizes, that are loosely arranged. This is evident in Figure 5B, in which the porosity percentage, determined using the liquid displacement method, was higher in GE_HA, followed by GE, compared to GE_HA_GNP and GE_GNP. The crosslinked hydrogels (GE_GNP and GE_HA_GNP), on the other hand, show a significant reduction in pore size, with them being denser and more compact, likely due to the formation of additional crosslinks between polymer chains inducing an increase in network compactness [24].

### 3.5. Physicochemical Characterization of the Hydrogel Patches

**Figure 6:**
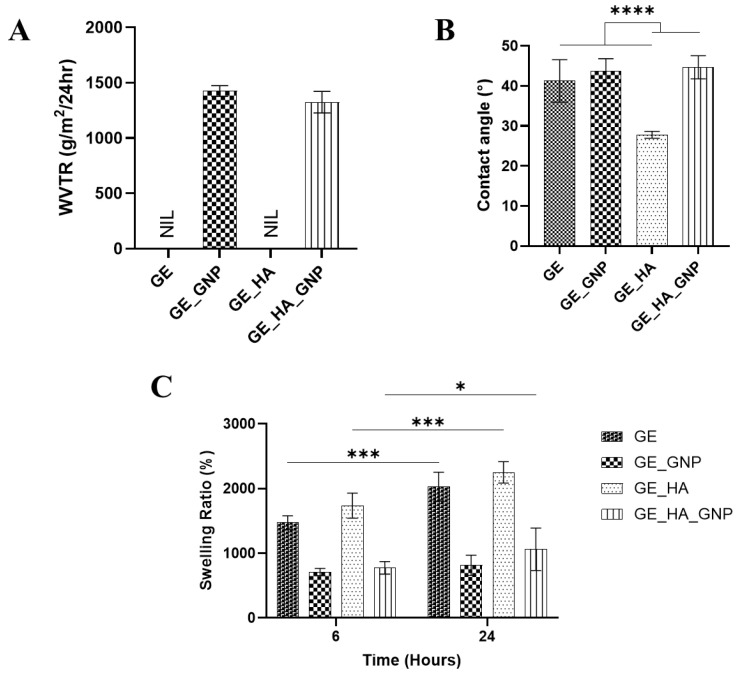
Physicochemical characterization of the hydrogel groups. (**A**) Water vapor transmission rate (WVTR) (g/m^2^/24 h), (**B**) contact angle (°), and (**C**) swelling ratio (%). * indicates a significance of *p* ≤ 0.05, *** indicates a significance of *p* ≤ 0.001, and **** indicates a significance of *p* ≤ 0.0001.

One of the important characteristics of a hydrogel is its ability to maintain water. The hydrogel patch is designed to maintain a moist environment and allow the water vapor to pass through the wound, facilitating exchange between the external and internal environment of the wound. A moist wound environment has several benefits in the acceleration of wound healing, including scar reduction, the promotion of cell migration, specifically keratinocytes, reducing inflammation, and many more [4]. The measurement of water vapor transmission rate (WVTR) is crucial in identifying the best formulation that fits all of the criteria to be a wound dressing, as it can directly influence the wound hydration level. A hydrogel dressing or a patch with a low WVTR can help to minimize water loss whereas a higher level of WVTR increases it. Selecting a patch with an optimal WVTR is very important, as it should not be so high that the wound dries out but, at the same time, it should not be so low as to cause excess wound fluid to accumulate. Previous studies have been performed using similar methods, and it was discovered that the ideal WVTR is typically below 1500 g/m^2^/24 h [4,16,25]. Furthermore, different wounds have different moisture requirements; for normal human skin, it is 204 ± 12 g/m^2^/24 h, but in first-degree burns, it is g/m^2^/24 h, and in skin injuries, it is 5138 ± 202 g/m^2^/24 h. The authors of previous studies have stated that acute wounds tend to have a significantly higher WVTR during the first 24–48 h compared to other stages of wounds. Thus, our crosslinked hydrogels, as seen in Figure 6A, were able to stay within the acceptable ranges of 500 to 1500 g/m^2^/24 h, whereby the highest WVTR was for GE_GNP with a value of 1426 ± 48.18 g/m^2^/24 h, followed by GE_HA_GNP with a value of 1324.42 ± 97.47 g/m^2^/24 h; this suggests that the combination of HA and GNP resulted in a denser hydrogel matrix. In contrast, for the non-crosslinked hydrogels, all were degraded completely within less than an hour.

Moving on to the analysis of contact angle, this analysis is important for assessing the hydrogels’ hydrophilic or hydrophobic nature on the surface. Generally, contact angles above 90° indicate a hydrophobic surface, whereas a low contact angle indicates a hydrophilic surface. A low contact angle is what is strived for in hydrogel patches since it can help to maintain a moist environment and encourage the growth of cell attachment. As observed in Figure 6B, the contact angles of all hydrogel groups are below 90°, indicating that these hydrogel formulations are all hydrophilic. The non-crosslinked hydrogels have a lower contact angle compared to the crosslinked hydrogels, although GE_HA (27.78 ± 0.854°) exhibited the lowest compared to GE (41.26 ± 5.31°). This may be due to HA being a naturally occurring polysaccharide that has a strong affinity towards water molecules, enhancing the hydrogel’s overall hydrophilicity [26]. The crosslinked patches, however, have a slightly higher contact angle than the non-crosslinked patches, wherein that of GE_HA_GNP (44.66 ± 2.88°) is the highest compared to GE_GNP (43.78 ± 2.61°), which indicates that HA does not significantly influence the surface wettability even with the addition of the crosslinker.

Another important criterion in a hydrogel patch is its ability to absorb water; by performing a swelling ratio analysis, this enables us to see whether the hydrogel patch can absorb exudate over time. The swelling ratio was observed and evaluated at 6 and 24 h to assess the hydrogel patches’ ability to absorb and retain moisture over a short and extended duration, mimicking real-case scenarios of the patient’s wound healing conditions. As shown in Figure 6C, GE exhibited a swelling ratio of 1473.91 ± 10.79%, which increased further to 2030.26 ± 22.11% at 24 h. Similarly, GE_HA displayed the highest swelling capacity among the tested groups, with values of 1737.96 ± 19.19% at 6 h and 2251.3 ± 16.175% at 24 h, aligning with the results of contact angle. This might be due to the inherent hydrophilic nature of hyaluronic acid (HA), which enhances water absorption and retention within the hydrogel matrix. In contrast, the crosslinked hydrogels demonstrated a significantly lower swelling ratio, indicating that the degree of crosslinking influences the reduction in the hydrogels’ expansion ability. GE_GNP exhibited a reduced swelling ratio of 710.30 ± 54.78% at 6 h, increasing to 815 ± 56.04% at 24 h. Similarly, the GE_HA_GNP hydrogel showed a swelling ratio of 775.225 ± 35.81% at 6 h and 1063.36 ± 32.125% at 24 h. According to previous studies, the optimum swelling properties of a hydrogel are reported to be around 500% [17]. However, other research suggests that it can vary between 500–1000% too, depending on the application of the hydrogel [27]. In this case, both GE and GE_HA_GNP are suitable for wound healing applications.

### 3.6. Mechanical Characterization of the Hydrogel Patches

#### 3.6.1. Physical Properties of Hydrogel Patches

**Figure 7:**
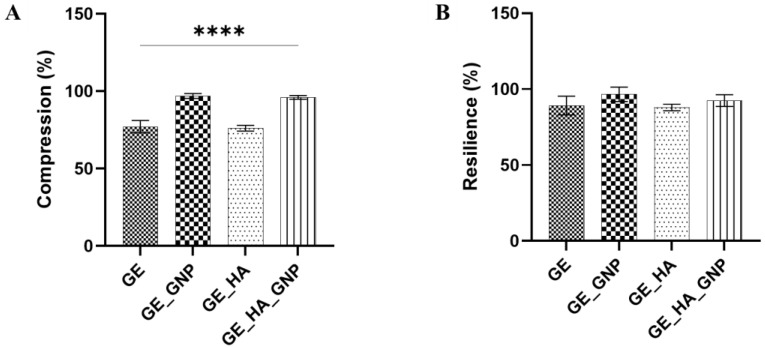
Mechanical characterization of the hydrogel groups. (**A**) Compression (%) and (**B**) resilience (%). **** indicates a significance of *p* ≤ 0.0001.

Mechanical testing, specifically through compression and resilience, is important for assessing a hydrogel’s durability. In terms of a hydrogel patch, compression analysis evaluates the hydrogels durability when facing external pressure, such as body movements. While resilience was observed for its ability to return to its original shape after deformation. Based on the mechanical testing results, both compression and resilience varied across the different hydrogel formulations, with the incorporation of GNP and HA significantly influencing the mechanical properties. Figure 7A shows that the highest compression percentage was recorded for GE_GNP (96.32 ± 1.53%) followed by GE_HA_GNP (95.97 ± 5.47%), suggesting that the crosslinker effectively enhances the hydrogel’s ability to withstand pressure. This aligns with previous findings that crosslinked hydrogels exhibit significantly higher compression ratios compared to non-crosslinked hydrogels, strengthening the mechanical robustness [18]. In contrast, the non-crosslinked GE (77.10 ± 4.68%) and GE_HA (76.06 ± 4.36%) hydrogels displayed lower compression, indicating a less rigid structure.

For resilience, as observed in Figure 7B, all hydrogels demonstrated good shape recovery after compression, with GE_GNP (96.54 ± 4.77%) and GE_HA_GNP (89.42 ± 5.16%) maintaining high resilience. The non-crosslinked hydrogels, GE (89.18 ± 5.54%) and GE_HA (86.19 ± 4.98%), had slightly lower resilience, suggesting that crosslinking enhances the hydrogel’s ability even more to return to its original shape after deformation. This observation is consistent with previous research indicating that crosslinked hydrogels, particularly those incorporated with genipin, exhibit superior resilience due to the reduced free amine groups and stronger intermolecular interactions.

#### 3.6.2. Tensile Properties Evaluation

**Figure 8:**
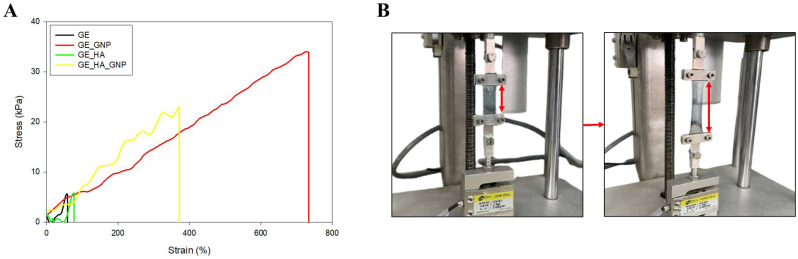
(**A**) Tensile stress–strain curves of all hydrogel groups (GE, GE_GNP, GE_HA, and GE_HA_GNP). (**B**) A before and after diagram of the tensile test using a universal tensile machine, where the red arrows indicate the elongation of the hydrogel samples, illustrating their deformation under tensile loading.

Tensile strength analysis of the hydrogels was conducted to observe the stretchability, elasticity, and robustness of the patches. This evaluation is important in understanding the performance of hydrogel since such patches will often be used in areas with frequent movement, such as in between joints. Based on Table 4 and Figure 8A, it can be seen that the crosslinked formulations, especially GE_GNP, exhibited the highest tensile strength (33.87 ± 9.58 kPa), followed by GE_HA_GNP (23.01 ± 6.83 kPa); this is most likely due to the incorporation of genipin into the hydrogel formulation, which enhances the mechanical robustness and strength of the polymer network. The GE_GNP formulation also shows a high elongation percentage of 733.5 ± 2.29%, indicating that the hydrogel is highly stretchable. On the other hand, the elongation of GE_HA_GNP is slightly lower at 371.62 ± 1.25%. On the other hand, non-crosslinked hydrogels such as GE served as a baseline with a tensile strength of 5.88 ± 1.46 kPa and elongation of 58.11 ± 0.51%. GE-HA also yields similar results to GE but with a slight increase in elongation of 76.87 ± 0.55%.

Other than the importance of adhesiveness, Table 4 shows the mechanical properties of each group. The incorporation of hyaluronic acid and genipin not only improved the mechanical strength but also enhanced the stretchability rate of the hydrogels. GE_GNP shows superior stretchability, followed by GE_HA_GNP, indicating that genipin plays a crucial role in strengthening the hydrogel network, while hyaluronic acid contributes to an increase in elastic properties due to it being a highly hydrophilic biopolymer. However, in this study, the shape used for the tensile analysis was rectangular-shaped, as seen in Figure 8B, but a dumbbell-shaped hydrogel with a short gauge length would have been preferred to provide a more uniform test and reduce the risk of stress concentration near the clamps [28]. In conclusion, while rectangular specimens were used in this study for simplicity and comparison purposes, adopting dumbbell-shaped specimens in future research will likely enhance the accuracy and reliability of mechanical property measurements, especially for hydrogels with lower mechanical strength or greater flexibility. This adjustment could help align the results with standardised testing protocols in the field.

## 4. Conclusions

After thorough fabrication and optimization of the topical hydrogel patch, the results showed that the incorporation of hyaluronic acid and genipin into a gelatin-based hydrogel significantly improved its mechanical strength and enhanced its properties. GEL_HA_GNP shows a balance in all properties, including effective moisture retention, thermal stability, and balanced swelling properties. This makes the patch an ideal and promising candidate for atopic dermatitis treatment, wound healing applications, or as a base material for future incorporations with active compounds, providing a more versatile base for advanced wound healing and regenerative medicine. Further studies should focus on in vitro studies and the antimicrobial properties of the hydrogel patch.

## Figures and Tables

**Table 1:** Composition of the topical hydrogel patch in 10 mL of dH_2_O.

Formulation	GE (g)	HA (g)	GNP (g)
GE	0.9	-	-
GE_GNP	0.9	-	0.01
GE_HA	0.9	0.02	-
GE_HA_GNP	0.9	0.02	0.01

**Table 2:** Thickness, weight variation, and folding endurance of the different hydrogel patch formulations.

No.	Formulation	Thickness (mm)	Weight Variation (g)	Folding Endurance
1	GE	1.24 ± 0.05	0.52 ± 0.02	64 ± 4.66
2	GE_GNP	1.04 ± 0.11	0.53 ± 0.01	233 ± 5.80
3	GE_HA	1.18 ± 0.19	0.49 ± 0.02	53 ± 4.02
4	GE_HA_GNP	1.02 ± 0.13	0.46 ± 0.02	300 ± 10.31

**Table 3:** Elemental compositions of the hydrogels using EDX, with which the levels of carbon (C), oxygen (O), and nitrogen (N) are shown.

Sample	C (%)	O (%)	N (%)
GE	45.3 ± 1.25	30.8 ± 0.9	22.4 ± 1.75
GE_GNP	48.1 ± 1.36	30.33 ± 1.06	19.2 ± 1.93
GE_HA	46.95 ± 0.95	30.55 ± 0.8	21.0 ± 1.35
GE_HA_GNP	46.3 ± 1.05	31.3 ± 0.85	20.55 ± 1.45

**Table 4:** Mechanical properties of the hydrogel groups.

Hydrogel	Tensile Strength (kPa)	Elongation (%)
GE	5.88 ± 1.46	58.11 ± 0.51
GE_GNP	33.87 ± 9.58	733.5 ± 2.29
GE_HA	5.81 ± 2.23	76.87 ± 0.55
GE_HA_GNP	23.01 ± 6.83	371.62 ± 1.25

## Data Availability

The authors confirm that the data supporting the findings of this study are available within the article. Further inquiries can be directed to the corresponding author.

## Abbreviations

The following abbreviations are used in this manuscript:

WVTR	Water vapor transmission rate
SEM	Scanning electron microscopy
TGA	Thermogravimetric analysis
GE	Gelatin
HA	Hyaluronic acid
GNP	Genipin
